# Exploration of the usability quality attributes of mobile government services: a literature review

**DOI:** 10.7717/peerj-cs.1026

**Published:** 2022-07-28

**Authors:** Abdulla Jaafar Desmal, Suraya Hamid, Mohd Khalit Othman, Ali Zolait

**Affiliations:** 1 Faculty of Computer Science and Information Technology, Universiti Malaya, Kuala Lumpur, Malaysia; 2 Department of Information Systems, University of Bahrain, Sakhir, Bahrain

**Keywords:** E-services, Mobile government services, Electronic services, Usability, Mobile usability

## Abstract

This article investigates and analyzes the usability quality attributes of mobile government services. The lack of previous research in the area of mobile government service quality encourages the researchers of the current work to select the usability quality dimension, which is considered one of the most significant parts of the mobile government service quality framework. Using the systematic literature reviews in the area of usability in human-computer interaction and software design, the main attributes are extracted and analyzed to fit into the context of mobile government services. Five quality attributes of the usability dimensions are identified for evaluation of the quality of services of mobile government. These attributes are efficiency, satisfaction, memorability, error and compatibility. The present research proposes a model that can be used to evaluate the usability of mobile government services. The attributes were extracted according to the mobility features with consideration of the service category (Government-To-Citizens). By measuring the usability quality of the mGovernment portal by the electronic government agencies, it leads to understanding the degree of usability of the provided services from the public’s perspective.

## Introduction

The strategy of delivering electronic services to the public has developed during recent years and has been influenced by information technologies. The popularity of using mobile devices by most people creates an opportunity for the service providers to use the mobile as a smart channel to deliver the services with more flexibility (Lim, Widdows & Park, 2006; Negi, 2009; Wang & Lin, 2012). The flexible interactions between the service provider and clients are essential to enhance the virtual service environment, encouraging the government sectors to start transferring electronic government services (eGovernment) to be available on a mobile government (mGovernment) portal. The context of mGovernment is different from other online services in terms of the features of mobile devices, the service provider category, the type of clients, the strategy, and the services processing cycle (Aloudat et al., 2014). From this point of view, the concept of quality concerning mobile government services requires more attention to improve the delivery process through the portal (Al-Hubaishi, Ahmad & Hussain, 2017). The availability of government services at all times is encouraging end-users to perform their transactions online through mobile devices (Song & Guan, 2015). End-users expect a quick response from mobile government applications, including smart options for better interactions between them and the government service providers.

The government service providers’ essential responsibilities are to ensure that people can receive the services anytime, anywhere, with less effort. The standard government services’ categories include education, health, financial, insurance, the municipality, and bill payment services (Sá, Rocha & Pérez Cota, 2016). Citizens expect to receive government services of high quality (Song & Guan, 2015). The study of Sharma (2015) stated that there was more attention paid to the government service providers to ensure citizens could access information and services. With emerging information and communication technologies (ICTs) in government sectors, many results have influenced the delivery of services through different portals to break the boundaries, such as eGovernment and mGovernment (Aloudat et al., 2014). The main advantage of mGovernment is that the user can perform the services using a smart mobile connected with an internet connection which creates a better interactive portal compared to other government service portals. To ensure continuous use and a high level of satisfaction with mGovernment services, there is a need to conduct regular reviews on the services’ delivery procedures in terms of technical quality and user satisfaction. It can be said that the mGovernment services have lowered the cost of processing the services, ensuring the delivery of services on time, creating smart interactions, and meeting the users’ needs (Saadi, Ahmad & Hussain, 2017).

Chanana, Agrawal & Punia (2016) affirm the necessity of service quality in the online portals, mainly the mobile services, because such services must be designed with mobile usability features that influence end-users’ experience and strengthen aspects of efficiency, accessibility, privacy, and security. Al-Hubaishi, Ahmad & Hussain (2017) argued that the mGovernment portal required a unique service quality measurement scale due to its mobility features, which differ from other online services. Using different quality measurement scales (*i.e*., e-commerce, eGovernment) for mobile government services can lead to inaccurate results and a more complex measuring process due to the unique characteristics that differ mobile government services from other measurement scales. A study of Lu, Zhang & Wang (2009) stated that there is a lack of literature on mobile quality services, which is necessary to provide a compatible measurement scale that fits the environment of mobile quality. Akter, D’Ambra & Ray (2010) discussed the importance of an integrated model to evaluate the service quality of the mobile portals and found that the comprehensive service quality scale leads to a better investigation of the service delivery process’s status and increases the end-users’ satisfaction.

The mobile government platform’s service quality framework consists of a multi-dimensional structure that measures the performance of service delivered to end-users by identifying the users’ expectations for the services. This is equivalent to the root concept of service quality that is stated “as a form of attitude, related but not equivalent to satisfaction, that results from the comparison of expectations with the performance” (Parasuraman, Zeithaml & Berry, 1985, p. 15). Thus, the present research investigates the dimension of “usability,” which is considered a fundamental component of the mobile government service quality framework.

Usability is one of the main dimensions of the service quality model for various sectors, e-services, e-commerce, eGovernment, and mGovernment (Hyvärinen, Kaikkonen & Hiltunen, 2005; Venkatesh, Ramesh & Massey, 2003). To strengthen the service quality model of mGovernment, it is necessary to investigate the relevant dimensions that fit within the context of mobile government services. Evaluation of service delivery through the mGovernment portal has different characteristics and procedures to other online services. Usability is part of the service quality framework of mGovernment, which plays an essential role in ensuring the effectiveness and efficiency of the service processing and delivery cycle (Al-Hubaishi, Ahmad & Hussain, 2017). The next section discusses the theoretical base of concepts in the pool of the usability dimensions of mobile government service quality.

Mobile government is a unique service portal that requires a comprehensive and compatible service quality measurement scale that can measure the mobility features and provide accurate results (Al-Hubaishi, Ahmad & Hussain, 2017, 2018; Chanana, Agrawal & Punia, 2016). The main research question is: RQ: What relevant constructs form the usability factor to measure mobile government service?

The present research evaluates the usability quality attributes based on the government to citizen (GTC) service category. The following section discusses the theoretical base of concepts in the pool of the usability dimension of mobile government service quality.

## Theoretical background

### Service quality

The term ‘service quality’ has drawn the attention of scholars and practitioners due to its importance in improving an organizations’ performance (Reis, Pena & Lopes, 2003). It enhances the organization’s success by following up on its best practice (Roberts, Varki & Brodie, 2003). Service quality refers to an organization’s ability to meet or exceed customer expectations (Parasuraman, Zeithaml & Berry, 1988). The service quality is an overall judgment that generates the difference between customer expectations of the service and the perceived service. A study by Yang & Chen (2000) suggests that it is necessary to understand customers according to their service quality perceptions. The customer’s perception is the comparison of the customer’s expectations with the customer’s perceived service. Quality of service represents the relationship between the service provider and the service delivered to the customer (Parasuraman, 2002). With the development of the world’s business and organization sectors, service quality attracts more attention from different fields, and it gets a range of definitions and extensions of construct based on the requirements and environment for each field (Ojasalo, 2001; Roberts, Varki & Brodie, 2003; Svensson & Padin, 2012).

**Figure 1:**
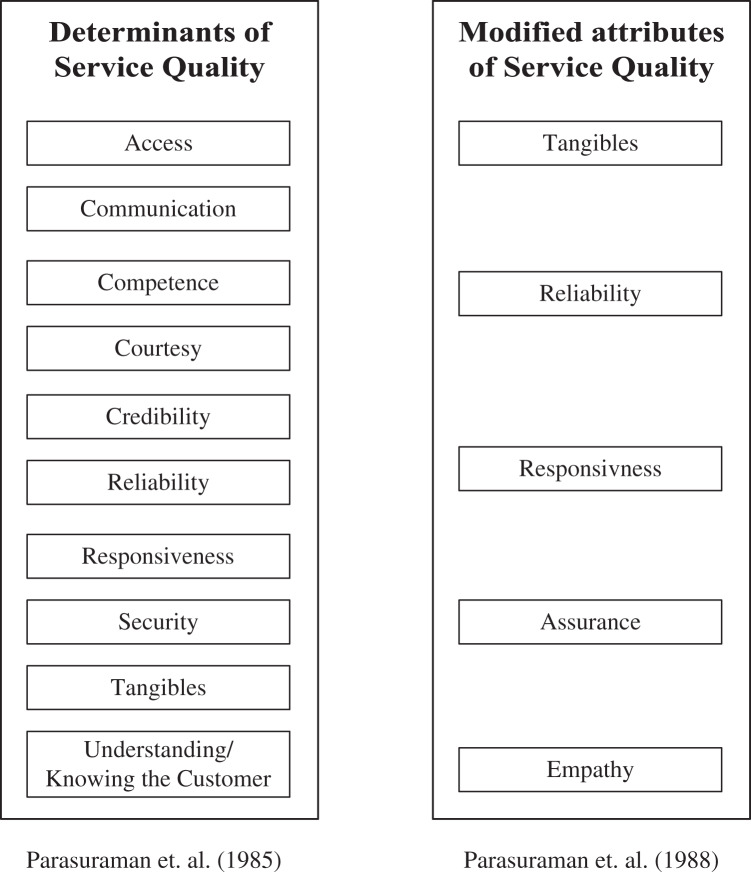
Original and modified version of SERVQUAL model.

Akter, D’Ambra & Ray (2010) stated that service quality research must be focused on a particular research environment, suggesting a need to identify basic concepts and measurements related to end-users’ perceptions, their detailed characteristics and the type of context. Categories of service quality dimensions differ among scholars. A study by Jaafar Desmal et al. (2019) stated that the two main dimensions of service quality are technical and functional, while Zhang et al. (2019) argue that service quality is constructed from eight main dimensions: website appearance, personal interaction, efficiency, aesthetic design, privacy/security, integration, personalization and fulfilment/reliability. The most famous theoretical base of service quality is the study by Parasuraman, Zeithaml & Berry (1988), which stated that the concept of service quality consists of 10 basic dimensions; these are “reliability, responsiveness, competence, access, courtesy, communication, credibility, security, understanding, and tangibles” These suggested ten dimensions have been reviewed and reduced to five main dimensions – “tangibles, reliability, responsiveness, assurance, and empathy” – and are referred to as the SERVQUAL quality model (see Fig. 1). Despite the popularity of the SERVQUAL model, it does not apply to all fields of services (Landrum & Prybutok, 2004). Many scholars have modified the mode of SERVQUAL. Cronin & Taylor (1992) have revised the SERVQUAL model and created a new model named SERVPERF that aims to measure service quality in the form of its performance (Lu, Zhang & Wang, 2009), while the model of SERVQUAL measures service quality in terms of both expectation and performance. Brady & Cronin (2001) suggested a service quality model consisting of three main dimensions: “interaction, physical experience, and outcome quality”. However, measuring the service quality required identifying related dimensions to the particular field of study to avoid failure or inaccurate evaluation results. Three main criteria need to be considered when measuring service quality: the objectives, the context of the research, and the type of service provided (Akter, D’Ambra & Ray, 2010).

The development of information and communication technologies (ICTs) has opened a way for businesses to integrate the technologies within self-service techniques. At the end of the 1990s, the research had started to focus on electronic service quality since running services were based on electronic transactions. The first definition of electronic service quality appeared in a study by Zeithaml, Parasuraman & Malhotra (2000) and was given as “the extent to which a website facilitates efficient and effective shopping, purchasing, and delivery”. This definition refers to the provider of services using a website to pay attention to customers’ needs and lessen the technical problems that may occur while performing electronic services. Since then, many scholars have proposed electronic service quality models. One of these is the study by Parasuraman, Zeithaml & Malhotra (2005) which proposed a model named “E-S-Qual” consisting of four attributes, these being “efficiency, fulfillment, system availability, and privacy”. Based on this e-SQ model, other scholars have proposed other models to fit the quality of e-commerce, websites, *etc*. The present research considers the theoretical models by Parasuraman, Zeithaml & Malhotra (2005) and Parasuraman, Zeithaml & Berry (1988) as guidelines to construct the usability quality model of the mGovernment portal.

In the field of mobile government services, a number of studies have analyzed the service quality framework. For example, Al-Hubaishi, Ahmad & Hussain (2017) conducted a study of the service quality framework of mobile government service. It proposed six dimensions: “interaction quality, environmental quality, information quality, system quality, network quality, and outcome quality”. Another study by Shareef et al. (2014) proposed a framework for mobile government services consisting of four main service quality dimensions: “interactivity, understandability, authenticity, and security”. The authors of Shareef et al. (2014) focused their exploratory study on the Indian mobile government’s service quality. They used an online survey to collect the data from a group of experts to prioritize the service quality parameters of Indian mGovernment services. The study of Chanana, Agrawal & Punia (2016) ranked the eighteen parameters of mGovernment service quality, and the top five parameters were “privacy, getting things done in the expected time frame, getting things done right the first time, ease of use of applications, and fast navigation through applications without jams”.

### Mobile government services

Mobile government is defined as “the use of various mobile platforms (*e.g*., cell phones, notepads) for deploying government information and services to citizens in a way that is independent of time and location” (Liu et al., 2014, p. 433). Mobile government is a form of government services provided to the public *via* mobile devices connected with a wireless internet connection (Saadi, Ahmad & Hussain, 2017). It is an upgraded electronic government service (Eom & Kim, 2014; Faisal & Talib, 2016). Mobile government services are not limited to mobile devices only, but it extends to all intelligent devices (Amailef & Lu, 2011). The accessibility from anywhere and at any time are the two fundamental unique characteristics of mobile government portals that encourage government authorities to extend the provided services through them. The mobile government environment has other unique characteristics, such as mobility, personalization and portability (Wang & Lin, 2012). With advanced wireless technologies and the popularity of using mobile devices, the government authorities can deliver governmental services to remote areas (Liu et al., 2014). The standard categories of mobile government are “Government To Government – G2G”, “Government To Employees – G2E”, “Government To Business – G2B”, and “Government to Citizens – G2C” (Amailef & Lu, 2011). This study analyzes the service quality delivered in the form of “Government to Citizens – G2C”.

### Concept of usability

The concept of usability is an absolute term driven from human-computer interaction (HCI). The definition of usability is the status of using the product or services by the targeted user to perform particular tasks with consideration of efficiency, effectiveness and satisfaction of use (ISO, 2011). The usability concept was reviewed and replaced with the term “user-friendly” in the early 1980s, which was ambiguous and reflected a subjective connotation (Issa & Isaias, 2015). The usability concept is illustrated in any product since the user can identify the detailed criteria of products effectively, efficiently, and whether they satisfy the user (Pant, 2015). Hence, the user can determine if the required product contains usability features (ChanLin & Hung, 2016). The three main features of a usable product are that a user can easily use a product for the first time, the product meets the users’ objectives, and the product provides an easy way to recall the interface and the ability to reuse it next time (ChanLin & Hung, 2016; Huang et al., 2015; Ke & Su, 2018). These three criteria differ among scholars. Authors Sagar & Saha (2017) argue that the standard usability criteria attributes involve “Efficiency, Effectiveness, Satisfaction, and Learnability”. The usability concept criteria are aimed at assessing a product by recording the user’s performance with the product (Wallace et al., 2013). Other measurement criteria of usability focus on the task, where tasks are divided based on sub-usability measurement criteria (Grönroos, 1984; Ke & Su, 2018; Wei, Chang & Cheng, 2015). Different applied criteria in the usability context enhance more detailed quantitative indicators that target improving productivity (ChanLin & Hung, 2016). Restrictions of usability criteria were found on measuring satisfaction and engagement due to being related to human emotion. Therefore, evaluating the usability of a system that requires human interaction needs to be scaled according to the domains of “efficient, effective, safe, utility, easy to learn, easy to remember, easy to use” (Issa & Isaias, 2015, p. 19). This is a core role of HCI when assessing the domain of the usability of electronic systems.

### Usability challenges and mobile government service quality

The mobile device with a wireless internet connection has unique characteristics that differ from computer devices. These characteristics pose challenges for scholars and participants when examining service quality usability dimensions in a mobile government context. Mobile devices’ special features involve mobility, connectivity, resolution of the display, limited screen size, limited power and processing speed, and limited input methods (Weichbroth, 2020).

– *Mobile context:* refers to the information that forms the interactions between the user, the application, and the surrounding environment. It may contain the location, objects, identities of people, and any elements that can affect the users’ attention (Ke & Su, 2018).
– *Connectivity:* A poor wireless connection is a common technical issue that is faced by general mobile users. This may occur while users are trying to access mGovernment services, or uploading or downloading files. The strength of the wireless data differs according to the location, time, and service provider. Therefore, the question of the connectivity of mobile government services must be considered while measuring service quality (Punchoojit & Hongwarittorrn, 2017).
– *The screen’s limited size:* A mobile device comes with a limited screen size, which impacts the usability dimension of the mGovernment service quality. Usability can measure the complexity of the contents of the mGovernment application and how it can be managed to fit with the end-users’ general devices
– *Display resolutions:* The resolution of the mobile’s screen size varies according to the range of pixels (480 × 800–1,440 × 2,560). This depends on the features of each brand of mobile device. It impacts the information illustrated as multimedia, which influences the usability of the mGovernment service (Zhang & Adipat, 2005).
– *Limited processing speed and power capacity:* The limited capacity for both the power and memory of mobile devices affects the applications’ processing speed, mainly when the running applications are based on graphics. Mobile devices are very slow when processing these. The limitations in speed and power capacity have influenced mobile government services (Sagar & Saha, 2017).
– *Methods of data entry:* Inputting the data into a small screen is challenging for service providers due to the screen size limitation. The means of inputting data into a mobile device can be through small buttons and a list of labels that allows end-users to select the required option. These input data methods are time-consuming and affect the speed of performing online transactions, especially when there is a time restriction to complete a particular task. Limitations of inputting data through mobile devices affects service quality in the form of usability (Wallace et al., 2013).

### Usability model of mobile government service quality

This study investigates the constructs related to usability dimensions, which are fundamental components in the mobile government framework. Few scholars have focused on the quality framework of mobile government services. Al-Hubaishi, Ahmad & Hussain (2017) and Shareef et al. (2014), have proposed service quality frameworks and suggested conducting further investigation into their dimensions and constructs. The majority of scholars have used other scale models to evaluate mobile service quality (Negi, 2009). Using other scales related to the mobile government’s environment makes it more complex and challenging to evaluate it due to the different contexts (Lu, Zhang & Wang, 2009; Wang & Lin, 2012). The absence of a particular scale for mobile government services leads organizations to use an incompatible service quality scale that gives the wrong service quality assessment (Özer, Argan & Argan, 2013).

Investigating the usability features for mGovernment portal required understanding the uniqueness of the smart devices that reflects on the end-users needs to deal and perform the transactions at mGovernment portal (Al-Hubaishi, Ahmad & Hussain, 2018; Alhammad & Elmouzan, 2020). Lu, Zhang & Wang (2009) considered the unique features of mobile services to propose a multidimensional, hierarchical model for measuring service quality. The study constructed a framework based on three dimensions that formed the model of service quality: interaction, environment and outcome quality. These fundamental dimensions are similar to studies by Cronin & Taylor (1992) and Rust & Oliver (1994). Venkatesh, Ramesh & Massey (2003) aimed to measure usability by comparing it when operating on both wired and wireless websites. The authors used the field study to investigate the usability of end-users’ perceptions while using the different websites. The data was collected from a total of 812 participates through a questionnaire. The websites fell into four categories: banking, tourism, news, and shopping, representing information and transactional websites. The results of the study found the usability attributes of mobile commerce to be “Content, Ease of use, Made-for-the medium, Promotion, and Emotion”. A study by Hyvärinen, Kaikkonen & Hiltunen (2005) conducted a usability test on two mobile banking applications and compared them in terms of navigation elements. The usability constructs proposed by this study were “Effectiveness, Efficiency & Satisfaction”. These usability constructs are derived from the International Organization for Standardization usability model (ISO 9241-11). Many scholars have investigated usability constructs based on the ISO model (Huang, Chou & Bias, 2005; Nah, Siau & Sheng, 2005; Wallace et al., 2013; Wei, Chang & Cheng, 2015). Other scholars have expanded the ISO usability model to include different constructs to cover the overall system and interface design of mobile applications (Cyr, Head & Ivanov, 2006; Gebauer, Tang & Baimai, 2008; Punchoojit & Hongwarittorrn, 2017). Recent scholars have investigated the usability construct to fit the context of electronic commerce, mobile commerce or electronic government. At the same time, there is a lack of studies investigating usability constructs that can be used to evaluate service quality in the mobile government context. In this section, the researcher proposes the usability constructs of the mobile government service quality framework.

#### Efficiency

Efficiency is an essential construct of usability service quality dimensions that refers to the user’s ability to perform a transaction with less time and increased accuracy. The definition of efficiency differs little among scholars. The authors’ first definition by Chittaro & Dal Cin (2002), Christie, Klein & Watters (2004), and Duh, Tan & Chen (2006) defined efficiency as the completion time of the task being performed by the user. The authors’ second definition, by Nourbakhsh et al. (2012) and Wei, Chang & Cheng (2015), defined it as the duration used to perform a transaction. The authors’ third definition of efficiency, by Cyr, Head & Ivanov (2006) and Wu & Wang (2005), defined it as the user’s duration on the screen for each transaction process. In mobile government services, the user needs to complete a task through a small device, a smartphone, that has limited input methods. Since the government sector provides the services, the user is required to prove their identification, upload documents or fill forms, all of which require more time in mobile government services than electronic government services, which has flexible input, a large screen and other related technologies. The efficiency in mobile government service requires more attention from scholars and participants to facilitate the technical characteristics of real transactions.

Based on the previous literatures, the present research conceptualizes the efficiency construct at measuring the usability of mobile government services. The user at mobile government expects to perform the service with less efforts that will reflects on the estimated time. The majority of the scholars relate the efficiency of service with estimated time, such as (Baashar et al., 2020; Li et al., 2020; Wang et al., 2019), and other scholars relate the efficiency with the unique characteristics of the smart devices such as (Dommaraju et al., 2022; Chan, Chiu & Ho, 2022; Rivo & Žumer, 2022). Hence, the efficiency quality of mobile government can be identified through estimated time to perform the service, the flexibility of input techniques, and the ability of user to move between the mobile service environment.

#### Satisfaction

This refers to the comfort level offered to end-users through using the software. Satisfaction reflects end-user attitudes toward software, these attitudes constructing the level of satisfaction (Nielsen et al., 2006). The usability measurement of ISO (2011) defines the concept of satisfaction as “reedom from discomfort, and positive attitudes towards the use of the product”. The main difference between the concepts of satisfaction in an electronic service environment and traditional services is the replacement of human-human interaction by human-machine interaction (Park & Joon Kim, 2013). A critical review of satisfaction with electronic service and, in particular, with mGovernment services ensures the end-users are comfortable with the transactional process. Abu-ELSamen et al. (2011) argue that the development of electronic services according to users’ needs increases satisfaction. The authors Jimenez, San-Martin & Azuela (2016) found a significant relationship between e-satisfaction and e-loyalty in constructing a “customer-level moderator” factor that impacts such services’ continued use. In mobile government services, the term satisfaction has a unique measurement scale that meets the service delivery mode through a mobile device. Deriving satisfaction criteria from other online services and applying them to the mGovernment context leads to more difficulties and conflicts since they are two different contexts. Each has its own characteristics based on the devices being used, the type of service provider (public, private), the objectives, and the service provider plan. The limitations of mobile devices may influence mobile government services’ continued use by the end-users since they expect a simple mobile application to perform their online transactions with greater satisfaction (Amin, Rezaei & Abolghasemi, 2014; Svendsen & Prebensen, 2013; Türkyılmaz & Özkan, 2007). Greater satisfaction with the mGovernment service encourages government authorities to provide more services using mobile platforms.

Measuring the satisfaction at the services based mobile devices must be formulated to reflect the characteristics of mobility, the uniqueness of smart devices, and the type of service being delivered to the end-users (Dommaraju et al., 2022; Hani et al., 2021; Xiong, Wang & Wang, 2022). These elements can guide the service provider to detect how the end-users are satisfied with the type of the services, the classifications of services, the support provided for mobile users, and ability of perform the entire service requirements through mobile devices.

#### Memorability

Memorability refers to the end-users’ ability to retain the process of using an application effectively (Kim & Jang, 2016). Some users do not use the mobile application regularly. In such cases, the end-users need to remember how to use the mobile application after a long period, and the providers need to ensure that the end-users can easily recognize the application without the need to relearn it (Fetscherin et al., 2015). Memorability can be measured by asking the end-users to compare their experience of using a particular mobile application between first-time use and regular use to find out how memorable the mobile application was. The end-users recalling their experience gives a sense of past experience which may impact the future behavior and motivation of the end-users (Kim, Ritchie & McCormick, 2012). Some factors that enhance memorability are construction of a simple mGovernment interface, the computability GUI of the mGovernment application with the e-government portal and minimizing the steps required to complete mGovernment services. A simple mGovernment service procedure can encourage all users with a different experience of online services to use this type of governmental service.

Identifying the memorability as a quality construct within the usability can construct a comprehensive service quality model for mobile government, that can enhance the service provider to facilitate the process of mobile government, the overall structure of the service, and the navigation within the service (Hani et al., 2021; Chan, Chiu & Ho, 2022; Rodriguez Müller et al., 2021; Soni et al., 2021). Another important criteria here is that the end-user be more familiar with the process of such category of the provided services, that get an advantage to increase the memorability and impact of usability of mobile government services.

#### Error

The concept of error has been used for usability scales by Nielsen et al. (2006) and Harrison, Flood & Duce (2013). The error in usability measurement refers to those errors made by users while using mobile applications. Identifying errors by end-users means providing certain notifications for developers to troubleshoot the end-users’ particular experience for more development in the future. The error attribute reflects how well the end-users are using mobile applications to perform the best quality transactions without technical errors. Nielsen et al. (2006) stated that end-users are making errors while using mobile applications, and there should be a quick recovery process to solve these errors. The rate of errors occurring is useful for developers to gauge the usability of the software. The PACMAD’s usability model contains error, measuring the error’s nature and frequency. Considering the concept of mobile government services, it is necessary to precisely measure the error to enhance current and future mGovernment services. Understanding errors leads to an improvement in the mobile application’s performance and accuracy.

#### Compatibility

The term compatibility refers to the ability of mobile application software to be run on the different operating systems of smart devices (iPhone, Android, Windows, *etc*.), with the consideration of minimal memory load for smart devices (Arvidsson, 2014; Lee & Lin, 2005; Wang, Cho & Denton, 2017). The attribute of compatibility ensures that the hardware and software of standard smart devices are performing their functions properly. Akhlaq & Ahmed (2013) argued that mobile compatibility ensures the validity of application behavior that meets the customers’ expectations. The attribute of mobile compatibility ensures that the contents fit on the screen of a smart device. Also, it ensures the appropriate navigation methods for smart devices are applied, the font sizes and objects are displayed appropriately on the application, and the functions and features meet the different operating systems of smart devices (Akhlaq & Ahmed, 2013; Murano, 2018). To get the best quality of mobile government services, it is necessary to ensure the smart devices are compatible in terms of software, hardware and data to enhance the users’ satisfaction and provide the best quality of mobile government services. Most previous studies measured mobile application usability without considering the relevant features when individuals held the mobile device (*i.e*., the mobile application’s ability to display the contents whether in vertical or horizontal mode, according to the position the device is being held by the end-user). The applications of the mGovernment portal must be compatible with the display mode of the devices to achieve user satisfaction.

As shown in Table 1, it summarized all of the recommended constructs to measure the usability quality at mobile government portal, that are efficiency, satisfaction, memorability, error, and compatibility.

**Table 1:** Usability’s attributes of mGovernment service quality.

Usability attribute	Supporting literature	Findings
Efficiency	(Burigat, Chittaro & Gabrielli, 2008)	Efficiency plays a fundamental role in enhancing the user experience of electronic services. It ensures that the user can use the software with ease and for less time to perform any transaction.
	(Christie, Klein & Watters, 2004)	
	(Duh, Tan & Chen, 2006)	
	(Harrison, Flood & Duce, 2013)	
	(ChanLin & Hung, 2016)	
	(Nourbakhsh et al., 2012)	
Satisfaction	(Abu-ELSamen et al., 2011)	It has been found that satisfaction with the services delivered through the mobile government platform can be achieved when it has been designed taking into consideration the mobile device’s limitations. This will ensure continued use of mGovernment services.
	(Park & Joon Kim, 2013)	
	(Padula & Busacca, 2005)	
	(Thakur, 2014)	
	(Shareef et al., 2014)	
	(Kim, Ritchie & McCormick, 2012)	
Memorability	(Kim, Ritchie & McCormick, 2012)	It has been found that memorability enhancement keeps users using mobile government services in an effective way. Some factors that enhance memorability are a simple construction of the mGovernment interface, the compatibility of the GUI of the mGovernment application with the e-government portal and minimizing of the steps needed to complete mGovernment services. In general, the mobile government authority can improve the memorability attribute for end-users by updating its factors regularly.
	(Fetscherin et al., 2015)	
	(Kim & Jang, 2016)	
	(Leung, McGrenere & Graf, 2011)	
	(Massey, Khatri & Ramesh, 2005)	
	(Harrison, Flood & Duce, 2013)	
	(Wei, Chang & Cheng, 2015)	
Error	(Nielsen et al., 2006)	It has been found that the error attribute can improve the current and future mobile government applications and keep in track with end-users’ comments. This attribute serves as an in-depth investigating of a mobile application from different users which helps developers to improve the application.
	(Harrison, Flood & Duce, 2013)	
	(Travis & Murano, 2014)	
	(Murano, 2018)	
	(Venkatesh, Ramesh & Massey, 2003)	
	(Wallace et al., 2013)	
Compatibility	(Lee & Lin, 2005)	It has been found that compatibility with smart devices in terms of software, hardware and data can enhance user satisfaction and offer the best quality of mobile government services.
	(Nusair & Kandampully, 2008)	
	(Akhlaq & Ahmed, 2013)	
	(Arvidsson, 2014)	
	(Wang, Cho & Denton, 2017)	

## Research methodology

### Search method

The present research uses literature reviews through the model of Okoli’s systematic steps (Okoli, 2015). The steps are planning, selection, extraction, and execution of literature reviews.

### E-Library databases

The electronic library databases that were used in the current research come from ScienceDirect, Scopus, Web of Science, SAGE, and ACM. The researchers set the period of published research starting from 2005–2022 to get more insight into the caller phone’s usability characteristics until the development of smartphones. The main search keywords were: “usability”, “mobile”, “mobile services”, “e-service”, “online service”, “electronic service”, “mobile government”, “electronic government”, “mGovernment”, “mGovernment service”, “service quality”, “e-service quality”, and “online service quality”. During the search of the e-library databases, the Boolean operators applied “AND”, “OR”, “NOT”, “SAME”, “NEAR” to get the most relevant literature.

### Research progress

The first stage of the literature review was the planning that aimed at identifying the research’s main purpose and clearly describe the detailed objectives. In this stage, the draft research protocol was agreed among the researchers to ensure the standard of research and output. The next step of the research progress was the selection process that acted as a screening for the inclusion of literature. The researchers agreed standard criteria to include or to eliminate literature. The inclusion of the literature was based on the use, evaluation or measuring of the concept of usability within the mobile device (cell phone or smartphone). The third stage was the extraction, which allowed the researchers to extract the data systematically and clearly described the detailed criteria of the usability according to the methodology used per study.

The last stage was the execution process which enabled the researchers to synthesize the collected literature and draw up the findings and write the results.

## Proposed usability model of mgovernment service quality

**Figure 2:**
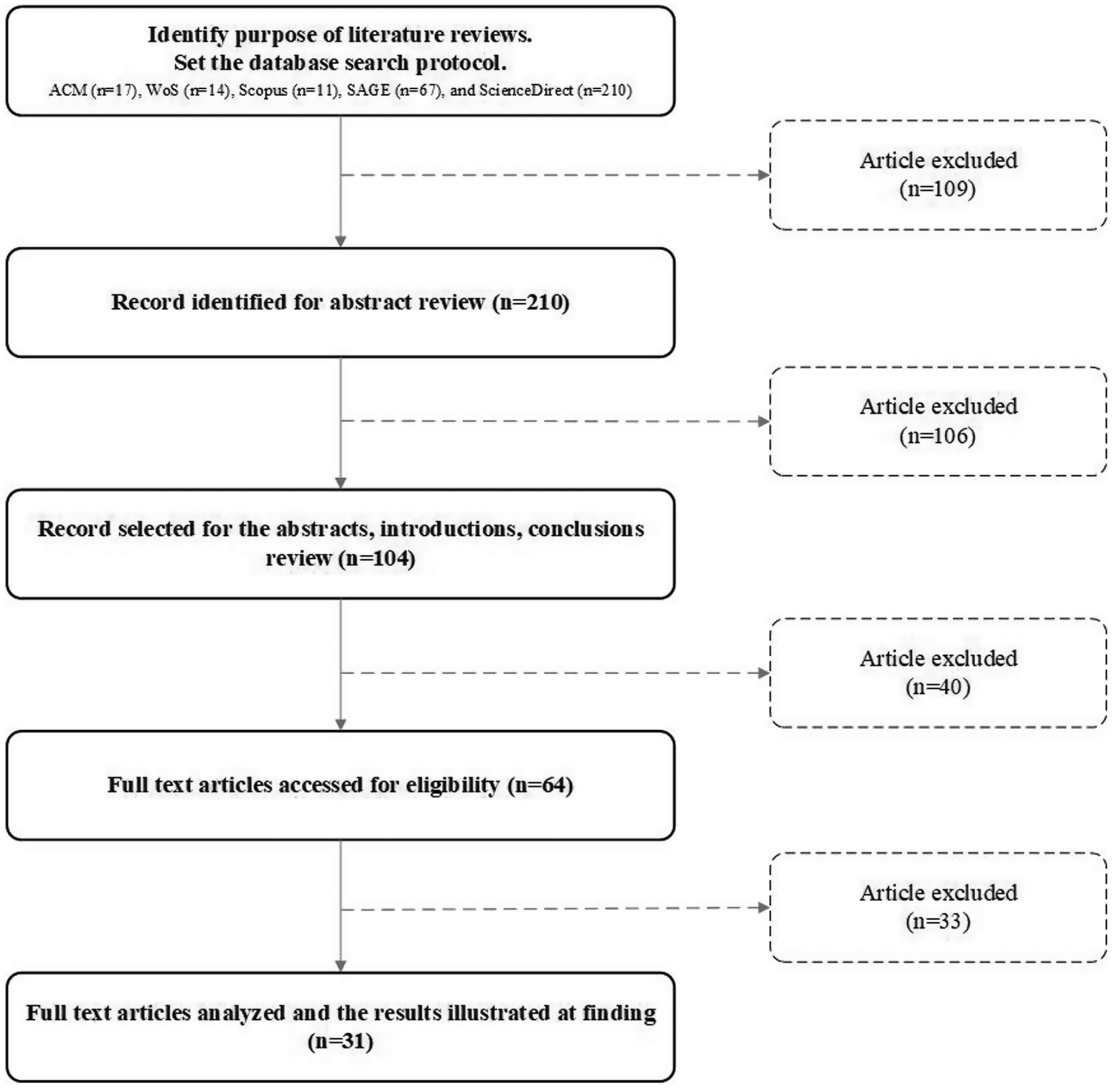
Systematic development for literature review.

**Figure 3:**
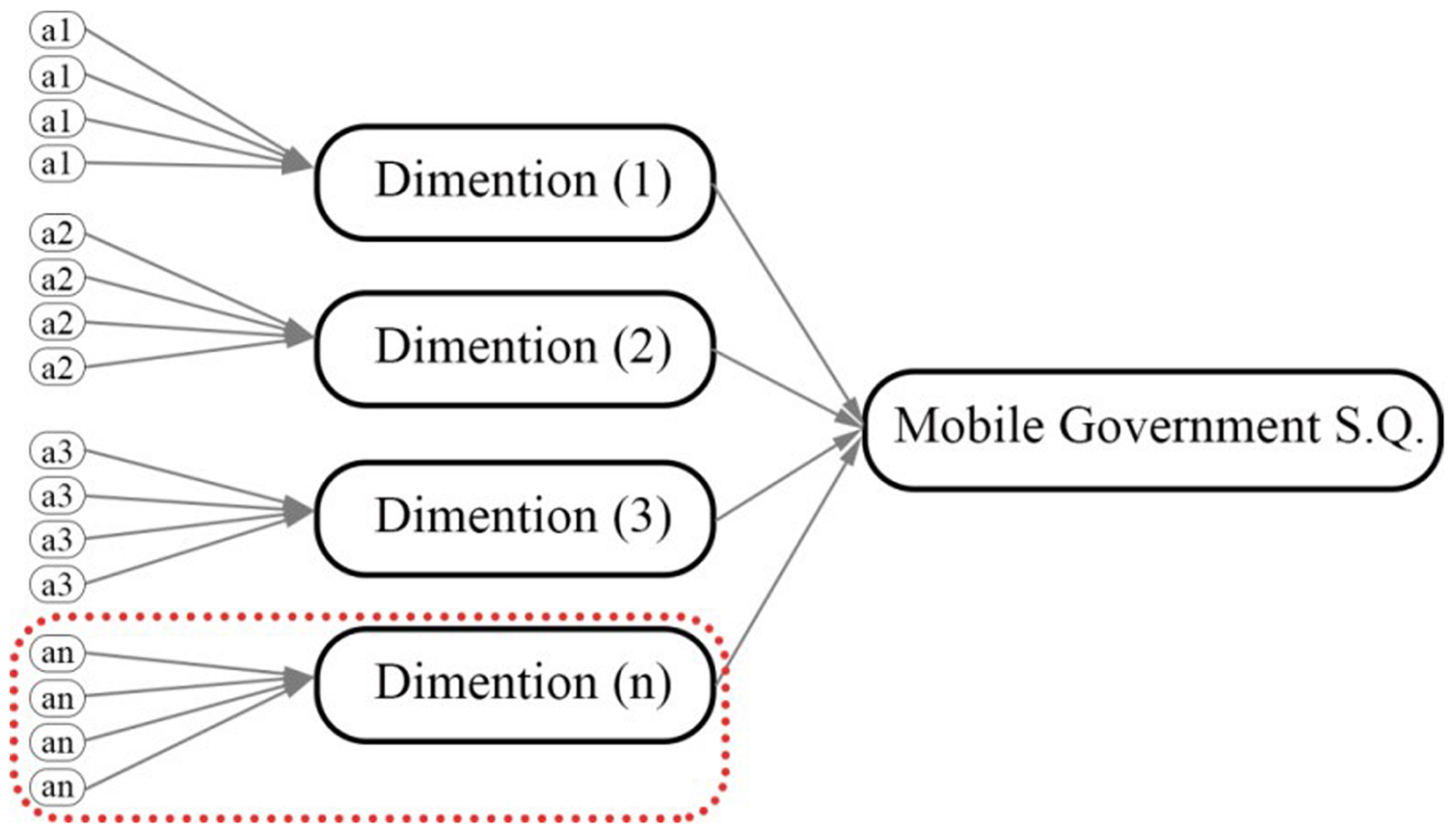
General overview of mGovernment framework with the selected part of the current research area.

**Figure 4:**
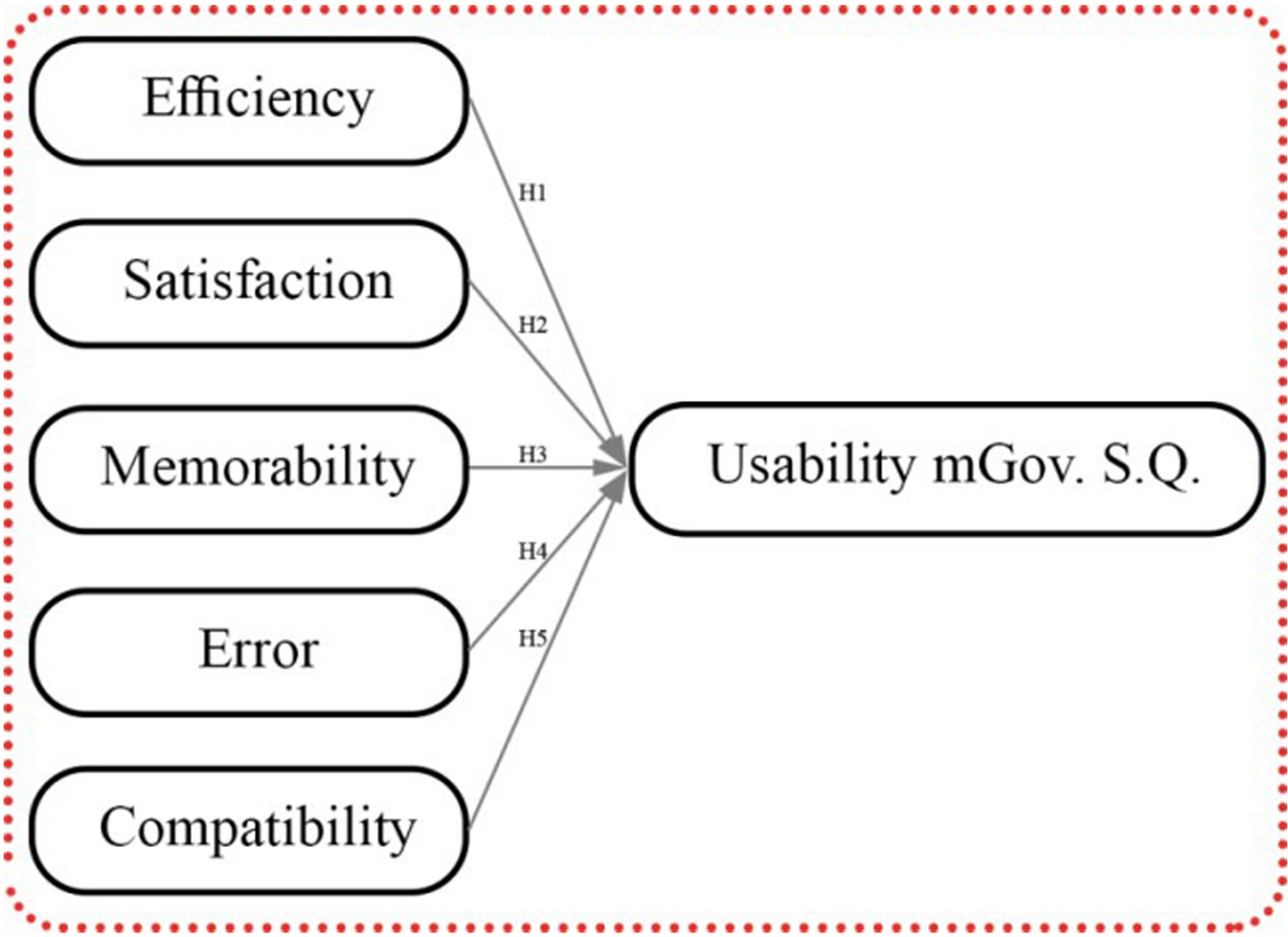
Current research area shows the proposed quality attributes of usability model at mGovernment service quality.

Five hypotheses are presented that construct a proposed model of the usability dimension for mobile government services. This research’s usability dimension refers to one component of the service quality framework of mGovernment services consisting of five proposed attributes belonging to the usability dimension. These proposed hypotheses are required to conduct empirical testing to find their impact on the context of the usability dimension of the mobile government context—a summary of the usability attributes mentioned in Table 1. A general overview of the mGovernment framework is presented in Fig. 2. A conceptual model of usability has been developed based on previous literature reviews, which illustrates the five service quality attributes that affect the context of mobile government services (see Fig. 3). The final results shows at the proposed quality attributes (see Fig. 4).

## Conclusion

Mobile Government is one of the modern technologies to deliver government services to the public. End users expect to perform their transactions with high-quality techniques, primarily when delivered through mobile devices, to ensure that these services are secured, high privacy, and designed as per end-user’s requirements. When mobile government services are designed and implemented with consideration of quality compatible with mGovernment portal, the government authority saves costs, efforts and ensures the continuous uses of such services. Due to the absence of detailed dimensions of the mGovernment framework, the current article investigated the usability dimension’s quality constructs that fit in the context of mGovernment services. The final results show five quality attributes that are measurable and compatible for the government mobility services that are: efficiency, satisfaction, memorability, error, and compatibility. The efficiency quality construct ensures that the end user can use the service qith easiest way, and less duration of time to perform the transaction. The satisfaction quality construct measures how the end user is satisfied with the functions provided that influence the continues use toward the service. The memorability quality construct measures the ability of end user to remember the steps to perform the services. The error quality construct measures the issues technical issues faced by end-user while performing the service at the mGovernment portal, while the compatibility quality construct measures how the service software has the compatibility features to be fit with the mobile device.

The results improved the ability of government authorities to consider these attributes while planning and designing the mGovernment service applications. However, the usability quality dimension is a fundamental part of mGovernment quality framework that works beside the other quality dimensions within the framework, such as interaction quality, efficiency quality, information quality, privacy, and security quality. Future research must investigate and analyze each dimension to identify the quality attributes within the mGovernment service environment.

## Research implications and future research

An in-depth investigation of the usability dimension has been applied in this research to provide a comprehensive understanding of related attributes to one of the leading mobile government framework components. Due to a lack of studies in mobile government service quality, this research adds value by providing a guideline for scholars and practitioners to collect detailed knowledge on the importance of usability for mobile government service. This encourages government authorities to improve mobile-based services to attract users, encouraging them to make more transactions through mobile government platforms. To continue improving the service quality at the mGovernment platform, future research must analyze other dimensions of the mGovernment service framework.

## Additional Information and Declarations

### Data Availability

The following information was supplied regarding data availability: This article is a literature review and does not contain raw data.

## References

[ref-1] Abu-ELSamen AA, Akroush MN, Al-Khawaldeh FM, Al-Shibly MS (2011). Towards an integrated model of customer service skills and customer loyalty. International Journal of Commerce and Management.

[ref-2] Akhlaq A, Ahmed E (2013). The effect of motivation on trust in the acceptance of internet banking in a low income country. International Journal of Bank Marketing.

[ref-3] Akter S, D’Ambra J, Ray P (2010). Service quality of mHealth platforms: development and validation of a hierarchical model using PLS. Electronic Markets.

[ref-4] Al-Hubaishi HS, Ahmad SZ, Hussain M (2017). Exploring mobile government from the service quality perspective. Journal of Enterprise Information Management.

[ref-5] Al-Hubaishi HS, Ahmad SZ, Hussain M (2018). Assessing M-government application service quality and customer satisfaction. Journal of Relationship Marketing.

[ref-6] Alhammad M, Elmouzan A (2020). Factors influencing Citizen’s adoption of M-government: the case of Saudi Arabia. Journal of Management and Strategy.

[ref-7] Aloudat A, Michael K, Chen X, Al-Debei MM (2014). Social acceptance of location-based mobile government services for emergency management. Telematics and Informatics.

[ref-8] Amailef K, Lu J (2011). A mobile-based emergency response system for intelligent M-government services. Journal of Enterprise Information Management.

[ref-9] Amin M, Rezaei S, Abolghasemi M (2014). User satisfaction with mobile websites: the impact of perceived usefulness (PU), perceived ease of use (PEOU) and trust. Nankai Business Review International.

[ref-10] Arvidsson N (2014). Consumer attitudes on mobile payment services—results from a proof of concept test. International Journal of Bank Marketing.

[ref-11] Baashar Y, Alhussian H, Patel A, Alkawsi G, Alzahrani AI, Alfarraj O, Hayder G (2020). Customer relationship management systems (CRMS) in the healthcare environment: a systematic literature review. Computer Standards & Interfaces.

[ref-12] Brady MK, Cronin JJ (2001). Some new thoughts on conceptualizing perceived service quality: a hierarchical approach. Journal of Marketing.

[ref-88] Burigat S, Chittaro L, Gabrielli S (2008). Navigation techniques for small-screen devices: an evaluation on maps and web pages. International Journal of Human Computer Studies.

[ref-13] Chan VHY, Chiu DKW, Ho KKW (2022). Mediating effects on the relationship between perceived service quality and public library app loyalty during the COVID-19 Era. Journal of Retailing and Consumer Services.

[ref-14] Chanana L, Agrawal R, Punia DK (2016). Service quality parameters for mobile government services in India. Global Business Review.

[ref-15] ChanLin L-J, Hung W-H (2016). Usability and evaluation of a library mobile web site. The Electronic Library.

[ref-16] Chittaro L, Dal Cin P (2002). Evaluating interface design choices on WAP phones: navigation and selection. Personal and Ubiquitous Computing.

[ref-17] Christie J, Klein RM, Watters C (2004). A comparison of simple hierarchy and grid metaphors for option layouts on small-size screens. International Journal of Human-Computer Studies.

[ref-18] Cronin JJ, Taylor SA (1992). Measuring service quality: a reexamination and extension. Journal of Marketing.

[ref-19] Cyr D, Head M, Ivanov A (2006). Design aesthetics leading to M-loyalty in mobile commerce. Information and Management.

[ref-20] Dommaraju SR, Robinson D, Khosla S, Pobee R, Del Rios M (2022). Challenges with text-based messaging platform to perform social needs assessments of patients presenting with COVID-19-like illness at an urban academic emergency department. Public Health in Practice.

[ref-21] Duh HB-L, Tan GCB, Chen VH (2006). Usability evaluation for mobile device: a comparison of laboratory and field tests.

[ref-22] Eom S-J, Kim JH (2014). The adoption of public smartphone applications in Korea: empirical analysis on maturity level and influential factors. Government Information Quarterly.

[ref-23] Faisal MN, Talib F (2016). E-government to M-government: a study in a developing economy. International Journal of Mobile Communications.

[ref-24] Fetscherin M, Diamantopoulos A, Chan A, Abbott R (2015). How are brand names of Chinese companies perceived by Americans?. Journal of Product & Brand Management.

[ref-25] Gebauer J, Tang Y, Baimai C (2008). User requirements of mobile technology: results from a content analysis of user reviews. Information Systems and E-Business Management.

[ref-26] Grönroos C (1984). A service quality model and its marketing implications. European Journal of Marketing.

[ref-27] Hani AB, Hijazein Y, Hadadin H, Jarkas AK, Al-Tamimi Z, Amarin M, Shatarat A, Abeeleh MA, Al-Taher R (2021). NC-ND license Cross-sectional Study E-Learning during COVID-19 pandemic; turning a crisis into opportunity: a cross-sectional study at the University of Jordan. Annals of Medicine and Surgery.

[ref-28] Harrison R, Flood D, Duce D (2013). Usability of mobile applications: literature review and rationale for a new usability model. Journal of Interaction Science.

[ref-29] Huang SC, Chou IF, Bias RG (2005). Empirical evaluation of a popular cellular phone’s menu system: theory meets practice. Journal of Usability Studies.

[ref-30] Huang Y-M, Pu Y-H, Chen T-S, Chiu P-S (2015). Development and evaluation of the mobile library service system success model. The Electronic Library.

[ref-31] Hyvärinen T, Kaikkonen A, Hiltunen M (2005). Placing links in mobile banking application.

[ref-32] ISO (2011). ISO/IEC/IEEE Systems and software engineering—architecture description. ISO/IEC/IEEE 42010:2011(E) (Revision of ISO/IEC 42010:2007 and IEEE Std 1471-2000).

[ref-33] Issa T, Isaias P (2015). Usability and human computer interaction (HCI). Sustainable Design.

[ref-34] Jaafar Desmal A, Khalit Othman M, Hamid S, Zolait A, Khalit Bin Othman M, Binti Hamid S, Hussain Zolait A (2019). Evaluation of mGovernment service quality from perspective of reliability.

[ref-35] Jimenez N, San-Martin S, Azuela JI (2016). Trust and satisfaction: the keys to client loyalty in mobile commerce. Academia Revista Latinoamericana de Administración.

[ref-36] Ke P, Su F (2018). Mediating effects of user experience usability. The Electronic Library.

[ref-37] Kim J-H, Jang SC (2016). Factors affecting memorability of service failures: a longitudinal analysis. International Journal of Contemporary Hospitality Management.

[ref-38] Kim J-H, Ritchie JRB, McCormick B (2012). Development of a scale to measure memorable tourism experiences. Journal of Travel Research.

[ref-39] Landrum H, Prybutok VR (2004). A service quality and success model for the information service industry. European Journal of Operational Research.

[ref-40] Lee G, Lin H (2005). Customer perceptions of E-service quality in online shopping. International Journal of Retail & Distribution Management.

[ref-91] Leung R, McGrenere J, Graf P (2011). Age-related differences in the initial usability of mobile device icons. Behaviour and Information Technology.

[ref-41] Li X, Zhao X, Xu W, Pu W (2020). Measuring ease of use of mobile applications in E-commerce retailing from the perspective of consumer online shopping behaviour patterns. Journal of Retailing and Consumer Services.

[ref-42] Lim H, Widdows R, Park J (2006). M—loyalty: winning strategies for mobile carriers. Journal of Consumer Marketing.

[ref-43] Liu Y, Li H, Kostakos V, Goncalves J, Hosio S, Hu F (2014). An empirical investigation of mobile government adoption in rural China: a case study in Zhejiang province. Government Information Quarterly.

[ref-44] Lu Y, Zhang L, Wang B (2009). A multidimensional and hierarchical model of mobile service quality. Electronic Commerce Research and Applications.

[ref-92] Massey AP, Khatri V, Ramesh V (2005). From the web to the wireless web: technology readiness and usability.

[ref-45] Murano P (2018). A new user interface for a text proofreading web portal in a digitization and crowdsourcing context. International Journal of Web Information Systems.

[ref-46] Nah FFH, Siau K, Sheng H (2005). The value of mobile applications: a utility company study. Communications of the ACM.

[ref-47] Negi R (2009). User’s perceived service quality of mobile communications: experience from Ethiopia. International Journal of Quality & Reliability Management.

[ref-48] Nielsen CM, Overgaard M, Pedersen MB, Stage J, Stenild S (2006). It’s worth the Hassle!: the added value of evaluating the usability of mobile systems in the field.

[ref-86] Nourbakhsh M, Zin RM, Irizarry J, Zolfagharian S, Gheisari M (2012). Mobile application prototype for on-site information management in construction industry. Engineering, Construction and Architectural Management.

[ref-94] Nusair K, Kandampully J (2008). The antecedents of customer satisfaction with online travel services: a conceptual model. European Business Review.

[ref-49] Ojasalo J (2001). Managing customer expectations in professional services. Managing Service Quality: An International Journal.

[ref-50] Okoli C (2015). A guide to conducting a standalone systematic literature review. Communications of the Association for Information Systems.

[ref-85] Özer A, Argan MT, Argan M (2013). The effect of mobile service quality dimensions on customer satisfaction. Procedia – Social and Behavioral Sciences.

[ref-89] Padula G, Busacca B (2005). The asymmetric impact of price‐attribute performance on overall price evaluation. International Journal of Service Industry Management.

[ref-51] Pant A (2015). Usability evaluation of an academic library website. The Electronic Library.

[ref-52] Parasuraman A (2002). Service quality and productivity: a synergistic perspective. Managing Service Quality: An International Journal.

[ref-53] Parasuraman A, Zeithaml VA, Berry L (1985). A conceptual model of service quality and its implications for future research. The Journal of Marketing.

[ref-54] Parasuraman AP, Zeithaml VA, Berry LL (1988). SERQUAL: a multiple-item scale for measuring consumer perceptions of service quality. Journal of Retailing.

[ref-55] Parasuraman A, Zeithaml VA, Malhotra A (2005). A conceptual framework for understanding E-service quality: implications for future research and managerial practice. Journal of Service Research.

[ref-56] Park E, Joon Kim K (2013). User acceptance of long-term evolution (LTE) services. Program.

[ref-57] Punchoojit L, Hongwarittorrn N (2017). Usability studies on mobile user interface design patterns: a systematic literature review. Advances in Human-Computer Interaction.

[ref-58] Reis D, Pena L, Lopes PA (2003). Customer satisfaction: the historical perspective. Management Decision.

[ref-59] Rivo K, Žumer M (2022). Academic libraries and use of mobile devices: case study of Slovenia. The Journal of Academic Librarianship.

[ref-60] Roberts K, Varki S, Brodie R (2003). Measuring the quality of relationships in consumer services: an empirical study. European Journal of Marketing.

[ref-61] Rodriguez Müller AP, Lerusse A, Steen T, Van de Walle S (2021). Understanding channel choice in users’ reporting behavior: evidence from a smart mobility case. Government Information Quarterly.

[ref-62] Rust RT, Oliver RL, Rust RT, Oliver RL (1994). Service quality: insights and managerial implication from the frontier. Service Quality: New Directions in Theory and Practice.

[ref-71] Sá F, Rocha Á, Pérez Cota M (2016). From the quality of traditional services to the quality of local E-Government online services: a literature review. Government Information Quarterly.

[ref-63] Saadi MR, Ahmad SZ, Hussain M (2017). Prioritization of citizens’ preferences for using mobile government services. Transforming Government: People, Process and Policy.

[ref-64] Sagar K, Saha A (2017). A systematic review of software usability studies. International Journal of Information Technology.

[ref-65] Shareef MA, Dwivedi YK, Stamati T, Williams MD (2014). SQ mGov: a comprehensive service-quality paradigm for mobile government. Information Systems Management.

[ref-66] Sharma SK (2015). Adoption of E-government services: the role of service quality dimensions and demographic variables. Transforming Government: People, Process and Policy.

[ref-67] Song M, Guan Y (2015). The electronic government performance of environmental protection administrations in Anhui province, China. Technological Forecasting and Social Change.

[ref-68] Soni P, Pal AK, Islam SKH, Singh A, Kumar P (2021). Provably secure and biometric-based secure access of E-Governance services using mobile devices. Journal of Information Security and Applications.

[ref-69] Svendsen GB, Prebensen NK (2013). The effect of brand on churn in the telecommunications sector. European Journal of Marketing.

[ref-70] Svensson G, Padin C (2012). Teleological approaches from complexity sciences in services. International Journal of Quality and Service Sciences.

[ref-90] Thakur R (2014). What keeps mobile banking customers loyal?. International Journal of Bank Marketing.

[ref-93] Travis C, Murano P (2014). A comparative study of the usability of touch-based and mouse-based interaction. International Journal of Pervasive Computing and Communications.

[ref-72] Türkyılmaz A, Özkan C (2007). Development of a customer satisfaction index model. Industrial Management & Data Systems.

[ref-73] Venkatesh V, Ramesh V, Massey AP (2003). Understanding usability in mobile commerce. Communications of the ACM.

[ref-74] Wallace S, Reid A, Clinciu D, Kang JS (2013). Culture and the importance of usability attributes. Information Technology & People.

[ref-75] Wang M, Cho S, Denton T (2017). The impact of personalization and compatibility with past experience on E-banking usage. International Journal of Bank Marketing.

[ref-76] Wang K, Lin CL (2012). The adoption of mobile value-added services: Investigating the influence of IS quality and perceived playfulness. Managing Service Quality.

[ref-77] Wang G, Lu R, Huang C, Guan YL (2019). An efficient and privacy-Preserving pre-clinical guide scheme for mobile eHealthcare. Journal of Information Security and Applications.

[ref-78] Wei Q, Chang Z, Cheng Q (2015). Usability study of the mobile library App: an example from Chongqing University. Library Hi Tech.

[ref-79] Weichbroth P (2020). Usability of mobile applications: a systematic literature study. IEEE Access.

[ref-87] Wu JH, Wang SC (2005). What drives mobile commerce? an empirical evaluation of the revised technology acceptance model. Information and Management.

[ref-80] Xiong L, Wang H, Wang C (2022). Predicting mobile government service continuance: a two-stage structural equation modeling-artificial neural network approach. Government Information Quarterly.

[ref-81] Yang H‐H, Chen KS (2000). A performance index approach to managing service quality. Managing Service Quality: An International Journal.

[ref-82] Zeithaml VA, Parasuraman A, Malhotra A (2000). A conceptual framework for understanding E-service quality: implications for future research and managerial practice (No. 00–115).

[ref-83] Zhang D, Adipat B (2005). Challenges, methodologies, and issues in the usability testing of mobile applications. International Journal of Human-Computer Interaction.

[ref-84] Zhang M, He X, Qin F, Fu W, He Z (2019). Service quality measurement for omni-channel retail: scale development and validation. Total Quality Management and Business Excellence.

